# Negative interference with antibody-dependent cellular cytotoxicity mediated by rituximab from its interactions with human serum proteins

**DOI:** 10.3389/fimmu.2023.1090898

**Published:** 2023-01-25

**Authors:** Saeko Yanaka, Rina Yogo, Hirokazu Yagi, Masayoshi Onitsuka, Natsumi Wakaizumi, Yuki Yamaguchi, Susumu Uchiyama, Koichi Kato

**Affiliations:** ^1^ Exploratory Research Center on Life and Living Systems (ExCELLS), National Institutes of Natural Sciences, Okazaki, Japan; ^2^ Institute for Molecular Science (IMS), National Institutes of Natural Sciences, Okazaki, Japan; ^3^ Graduate School of Pharmaceutical Sciences, Nagoya City University, Nagoya, Japan; ^4^ Graduate School of Pharmaceutical Sciences, Kyushu University, Fukuoka, Japan; ^5^ Graduate School of Technology, Industrial and Social Sciences, Tokushima University, Tokushima, Japan; ^6^ Graduate School of Engineering, University of Osaka, Osaka, Japan

**Keywords:** human serum albumin, therapeutic antibody, antibody-dependent cellular cytotoxicity, NMR, stable isotope labeling

## Abstract

Although interactions of small molecular drugs with serum proteins have been widely studied from pharmacokinetic and pharmacodynamic perspectives, there have been few reports on the effects of serum components on therapeutic antibody functions. This study reports the effect of abundant serum proteins on antibody-dependent cellular cytotoxicity (ADCC) mediated by rituximab and Fcγ receptor III (FcγRIII). Human serum albumin (HSA) and the Fab fragment from the pooled serum polyclonal IgG were found to compromise ADCC as non-competitive inhibitors. Our nuclear magnetic resonance data provided direct evidence for the interactions of HSA with both the Fab and Fc regions of rituximab and also with the extracellular region of FcγRIII (sFcγRIII). The degree of involvement in the interaction decreased in the order of rituximab-Fab > rituximab-Fc > sFcγRIII, suggesting preferential binding of HSA to net positively charged proteins. Although much less pronounced than the effect of HSA, polyclonal IgG-Fab specifically interacted with rituximab-Fc. The NMR data also showed that the serum protein interactions cover the Fc surface extensively, suggesting that they can act as pan-inhibitors against various Fc receptor-mediated functions and pharmacokinetics. Our findings highlight the importance of considering serum–protein interactions in the design and application of antibody-based drugs with increased efficacy and safety.

## 1 Introduction

Most drugs enter the blood after administration and are distributed to various tissues. In the bloodstream, they bind to serum proteins, such as albumin. The interactions of drugs with serum proteins significantly affect their pharmacokinetics and pharmacodynamics and are therefore carefully evaluated during drug discovery and development (1–3). Many of the currently used therapeutic antibodies are administered intravenously and exert their effect in the blood (4). However, limited studies have been conducted on the effects of interactions between therapeutic antibodies and serum proteins.

A previous study found that serum polyclonal antibodies had a negative effect on antibody-dependent cellular cytotoxicity (ADCC) (5). This may be the result of their competitive inhibition of the binding of therapeutic antibodies to Fcγ receptor IIIa (FcγRIIIa) through their Fc region. In this regard, the binding target of the serum protein is not the therapeutic antibody, but rather its cognate receptor. Such Fc-mediated competitive binding unavoidably occurs with various Fc receptors, including FcRn, thereby affecting other effector functions and pharmacokinetics of therapeutic antibodies, such as blood half-life (6). On the other hand, several studies have indicated that human serum components can interact with immunoglobulin G (IgG). For instance, analytical ultracentrifugation indicated that human serum albumin (HSA) interacts with human monoclonal IgG1 (7, 8), whereas our nuclear magnetic resonance (NMR) studies demonstrated that mouse IgG2b semi-specifically interacts with the Fab region of pooled human serum polyclonal antibody but barely with HSA (9, 10). However, the functional effects of these interactions remain unexplored.

Given this situation, we investigated the effects of the interaction between serum proteins and human IgG1 on its ADCC function mediated by FcγRIIIa. We used rituximab, an anti-CD20 mouse/human-chimeric IgG1 (11), along with HSA and polyclonal antibody, which together account for >70% of the serum proteins (12). Additionally, we characterized the interactions using stable-isotope-assisted NMR spectroscopy.

## 2 Materials and methods

### 2.1 Human serum proteins

Pooled off-the-clot human serum was purchased from Access Biologicals. Human serum polyclonal IgG and HSA were purchased from Sigma-Aldrich. The Fab fragment of the polyclonal IgG was digested using papain (13), purified using a Protein G Sepharose column (Cytiva) to remove Fc fragments, and then applied to a Superdex 200 16/60 gel filtration column (Cytiva) as previously described (9). Rituximab without isotope labeling was purchased from Zenyaku Kogyo.

### 2.2 Preparation of isotope-labeled proteins

Metabolic isotope labeling of antibodies was performed as previously described (14). A Chinese hamster ovary cell line producing rituximab (14), grown in a modified Nissui NYSF-404 medium containing appropriate stable isotope-labeled metabolic precursors was used. The amino acid components in the medium were substituted by uniformly ^15^N-labeled algal amino acid mixture supplemented with ^15^N-labeled analogs of the following amino acids: L-leucine, L-histidine, L-cysteine, and L-asparagine. The Fab and Fc fragments of isotope-labeled IgG1 were prepared through proteolytic digestion using papain as described above and subjected to NMR measurements. Digestion products were separated into Fab and Fc fragments using a protein A affinity column (GE Healthcare) and further purified through gel filtration using a HiLoad 16/60 Superdex 200 pg column (GE Healthcare) equilibrated with 50 mM Tris–HCl (pH 8.0) containing 150 mM NaCl (9). The rituximab-Fc fragment was treated with recombinant β1,4-galactosidase from *Streptococcus pneumoniae* (New England Biolabs), producing a uniformly fucosylated, nongalactosylated glycoform as previously described (14). A soluble form of human FcγRIIIb (NA2 form) composed of the extracellular domains (simply designated as sFcγRIIIb) was bacterially expressed with a C-terminal hexahistidine tag and uniform ^15^N-labeling and purified as previously described (15).

### 2.3 NMR measurements and spectral analysis

NMR measurements, the concentrations of the ^15^N-labeled forms of rituximab-Fc, rituximab-Fab, and sFcγRIIIb were set to 120 μM, 240 μM, and 120 μM, respectively, in 5 mM sodium phosphate buffer containing 50 mM NaCl. Serum polyclonal IgG, its Fab fragment, and HSA were added at final concentrations of 120 μM, 240 μM and 600 μM, respectively. The pH and temperature of the solutions were set to pH 7.4 and 37°C, respectively. ^1^H-^15^N heteronuclear single-quantum coherence (HSQC) peaks originating from the backbone of Fc were assigned based on the previous assignment in the BioMagResBank database (http://www.bmrb.wisc.edu) under the accession number 25224 (16). A series of NMR spectra were obtained using AVANCE 800 and AVANCEIII 900 spectrometers (Bruker BioSpin). The obtained data were processed using the NMRpipe software (17).

### 2.4 Biolayer interferometry analysis

An Octet HTX system (Sartorius) was used for biolayer interferometry (BLI) measurements of rituximab-FcγRIIIa interaction using the extracellular region of human FcγRIIIa (158V allele) in which Asn45 and Asn162 were N-glycosylated while the remaining N-glycosylation sites were substituted by glutamine. This bis-glycosylated receptor was expressed by CHO/dhFr- cells (ATCC^®^ CRL-9096) with a C-terminal hexahistidine tag, purified using cOmplete His-Tag Purification Resin column (Roche), and biotinylated as previously described (13, 18, 19). Before each assay, SAX (High Precision Streptavidin) biosensor tips were pre-wetted in 200 µL of HBS-P^+^ buffer (Cytiva, BR100827; 0.01 HEPES, 0.15 M NaCl and 0.05% (v/v) Surfactant P20) for at least 10 min. The measurements were performed at 30°C. First, a baseline was established using the buffer for 90 s, followed by the capture of the biotinylated receptor. Subsequently, a second baseline was obtained using HBS-P^+^, followed by the association and dissociation of rituximab with or without HSA and polyclonal IgG-Fab. The two-fold dilution series of rituximab starting at 2.0 µM were used for the assay. The regeneration step was performed using 1 M MgCl_2_ (Fujifilm Wako Pure Chemical Corp., 136–03995) after the cycle, and the same samples were measured thrice (*n* = 3).

### 2.5 ADCC reporter bioassay

Cell-based ADCC assays of rituximab were performed using a nuclear factor of activated T cell (NFAT)-driven luciferase reporter system (recombinant Jurkat T cells expressing firefly luciferase gene under the control of NFAT response elements with constitutive expression of human FcγRIIIa, high affinity (V158) variant and FcR γ chain) as previously described (20). Briefly, Jurkat/FcγRIIIa/NFAT-Luc cells (effector cells) were seeded at an effector with a target ratio of 10:1 and cultured with serially diluted rituximab mixed with polyclonal IgG-Fab or HSA at a final concentration of 240 μM or 600 μM, respectively. After incubation at 37°C for 4 h in a 5% CO_2_ atmosphere, luciferase activity was evaluated using the ONE-Glo Luciferase Assay System (Promega).

### 2.6 Protein isoelectric point calculation

Protein isoelectric point was calculated using Compute pI/Mw (21).

## 3 Results

**Figure 1:**
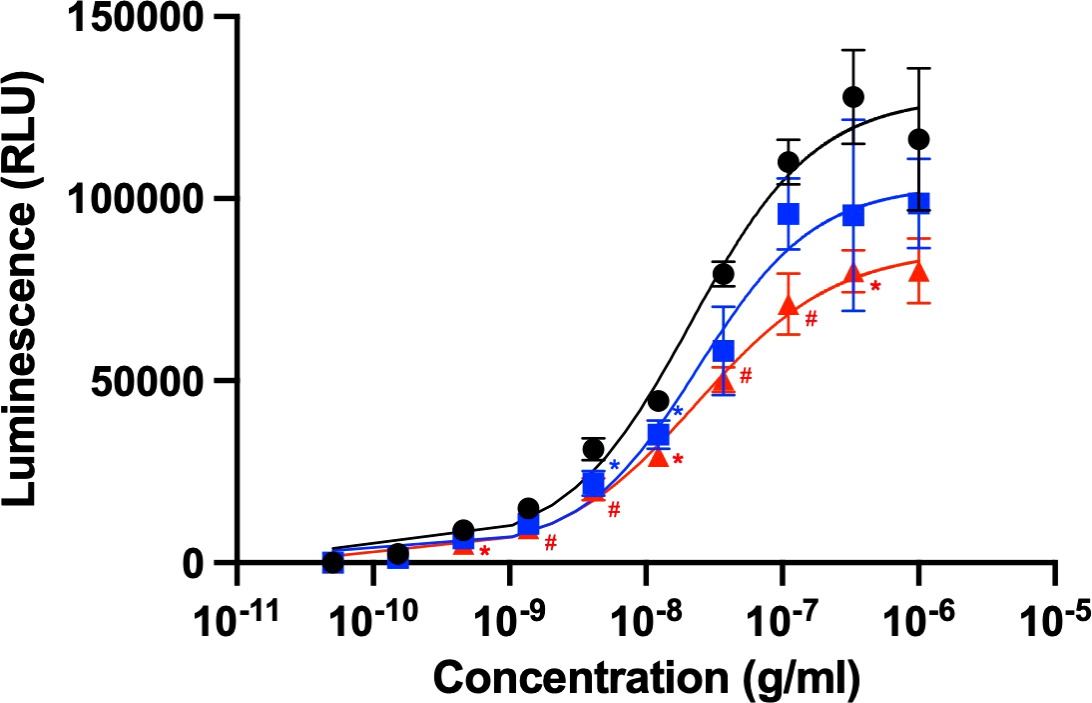
Rituximab-mediated ADCC activity influenced by the presence of serum components. ADCC activity of rituximab was measured in the absence (black circle) or presence of HSA (red triangle) or polyclonal IgG-Fab (blue rectangle). Rituximab concentration was prepared as a series of 3-fold dilutions from 1 μg/mL. Final concentrations of HSA and polyclonal IgG-Fab were 600 μM and 240 μM, respectively. Data represent the mean ± SD (n = 3). Significant *p*-values (*p < 0.05, ^#^ *p* < 0.01) are compared to the control by two-way ANOVA followed by Tukey’s multiple comparisons.

Rituximab is clinically used to treat certain cancers, including chronic lymphocytic leukemia and non-Hodgkin’s lymphoma (11). The anticancer activity of this therapeutic antibody is based on ADCC through its interaction with FcγRIIIa, which is typically expressed on natural killer cells. We evaluated ADCC activity using a reporter cell line expressing human FcγRIIIa and NFAT-driven luciferase reporter gene (20). The EC50 obtained from the present ADCC reporter assay was approximately 20 ng/mL, which is consistent with the previous report (22). The serum concentrations of HSA and polyclonal IgG have been reported as 35-50 mg/mL and 4.1-21.7 mg/mL, respectively (23, 24). Taking this into account, 600 μM of HSA or 240 μM of polyclonal human IgG-Fab were added to this cell-based assay system. However, an equimolar amount of their Fab fragment was used instead of polyclonal IgG antibodies because full-length IgG was expected to be competitive with rituximab regarding the Fc-mediated binding to FcγRIIIa (5). The assay results revealed that ADCC was moderately compromised by the polyclonal IgG-Fab fragments, while HSA negatively affected ADCC more intensely (**Figure 1**). These serum proteins little affected EC50, but rather reduced the magnitude of the maximum response, indicating their non-competitive inhibition.

**Figure 2:**
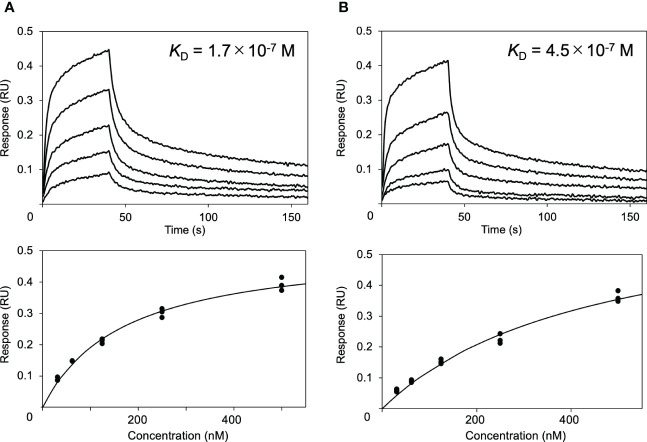
Effect of HSA on the interaction between rituximab and FcγRIIIa. Interaction of rituximab with the immobilized form of the extracellular region of human FcγRIIIa was observed by BLI in the **(A)** absence or **(B)** presence of HSA.

Next, the effects of these serum proteins on IgG1–FcγRIIIa interaction were examined *via* BLI analysis. The extracellular region of FcγRIIIa was immobilized onto a sensor, which was subjected to rituximab solutions in the presence or absence of HSA or polyclonal IgG-Fab. The results showed that HSA negatively affected their interaction, while no significant inhibition was observed by polyclonal IgG-Fab (**Figure 2**). Hence, we attempted to use a more sensitive technique for detecting weak interactions involving the serum proteins.

**Figure 3:**
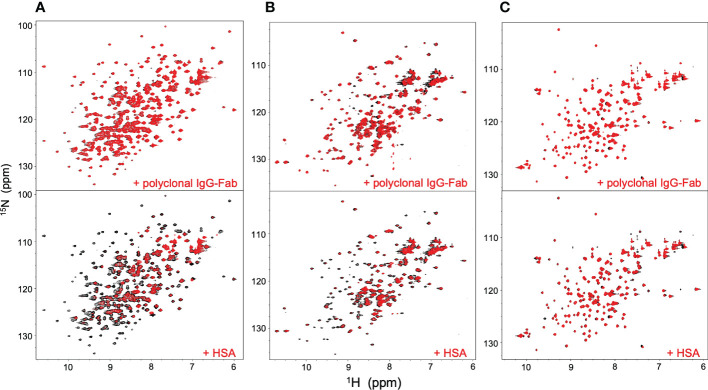
HSQC spectral changes of rituximab-Fc, rituximab-Fab, and sFcγRIIIb induced by the serum components. HSQC spectra of uniformly ^15^N-labeled rituximab-Fab **(A)**, rituximab-Fc **(B)**, and sFcγRIIIb **(C)** were measured in the absence (black) or presence (red) of HSA (lower) or polyclonal IgG-Fab (upper).

**Figure 4:**
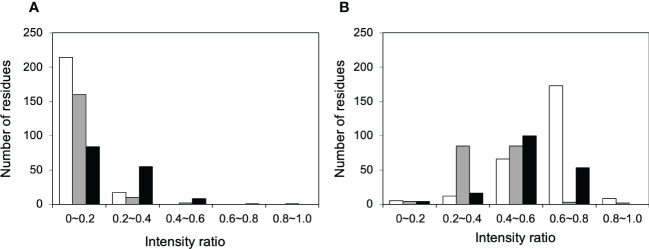
Histograms of the ^1^H-^15^N HSQC peaks exhibiting attenuation in intensity upon addition of serum proteins. The residues showing different degree of peak attenuation by the addition of **(A)** polyclonal IgG-Fab and **(B)** HSA were counted for ^15^N-labeled forms of rituximab-Fab (white), rituximab-Fc (black), and sFcγRIIIb (gray). The data were calculated from the spectra shown in **Figure 3**. The attenuation in intensity [(*I*o-*I*p)/*I*o, where *I*o and *I*p are original peak intensity and intensity after perturbation, respectively] was calculated for all observable ^1^H-^15^N HSQC peaks. The average levels of peak intensity attenuation caused by HSA were 0.60, 0.51, and 0.41 for rituximab-Fab, rituximab-Fc, and sFcγRIIIb, respectively, and those by polyclonal IgG-Fab are 0.33, 0.07, and 0.11 for rituximab-Fab, rituximab-Fc, and sFcγRIIIb, respectively.

**Figure 5:**
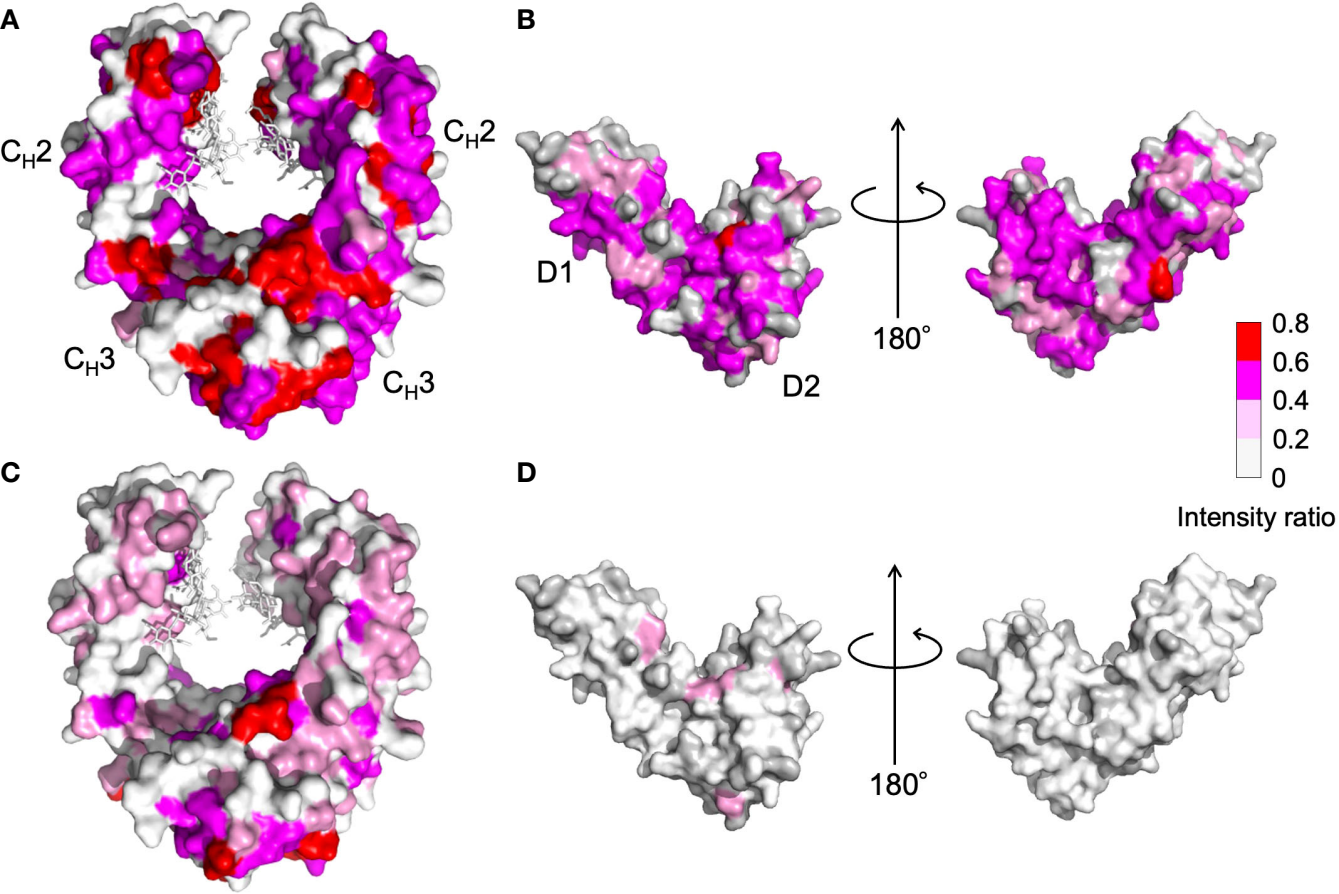
Mapping on the crystal structure of **(A, C)** human IgG1-Fc [PDB code: 3ave (27)] and **(B, D)** sFcγRIIIb [PDB code: 1T89 (28)] with the observed spectral perturbations by addition of **(A, B)** HSA, and **(C, D)** polyclonal IgG-Fab. The attenuation in intensity of the HSQC peaks originating from the backbones of human IgG1-Fc and sFcγRIIIb was calculated as (*I*o-*I*p)/*I*o, where *I*o and *I*p are original peak intensity and intensity after perturbation, respectively, and mapped on their crystal structures. The proline residues and the residues whose ^1^H-^15^N HSQC peaks could not be observed as probe because of broadening and/or overlapping are shown in gray. The N-glycans of IgG1-Fc are shown as stick models. The molecular graphics were generated using PyMOL (29).

**Figure 6:**
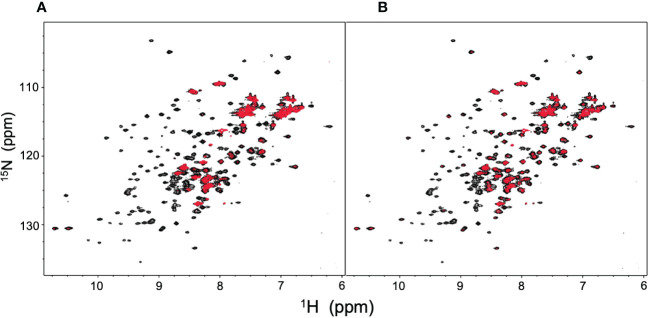
HSQC spectral changes of rituximab-Fc induced by human serum and human serum polyclonal IgG. HSQC spectra of uniformly ^15^N-labeled rituximab-Fc measured **(A)** in human serum or **(B)** in the presence of polyclonal IgG (red) are superposed on the spectrum of rituximab-Fc alone (black).

NMR can be a valuable tool for detecting weak interactions while dealing with heterogeneous systems. For selective observation of antibody NMR signals, we have established stable-isotope-labeling techniques of IgG glycoproteins using various eukaryotic expression systems (10, 14, 25, 26). In the present study, we prepared uniformly ^15^N-labeled rituximab, which was cleaved into Fab and Fc fragments for HSQC spectral measurements, to assess their interactions with HSA or polyclonal IgG-Fab. Additionally, we subjected uniformly ^15^N-labeled sFcγRIIIb to HSQC spectral measurements. Upon addition of HSA, the HSQC peaks of these ^15^N-labeled proteins exhibited attenuation of intensity (**Figure 3**), which was quantified for each peak according to the equation (*I*o-*I*p)/*I*o, where *I*o and *I*p are original peak intensity and intensity after perturbation, respectively. The degree of impact by HSA decreased in the order of rituximab-Fab > rituximab-Fc > sFcγRIIIb (**Figure 4**). The serum polyclonal IgG-Fab induced significant intensity attenuation for the peaks originating from rituximab-Fc, but the impact was smaller than that caused by HSA (**Figures 3**, **4**). We have established assignments of the backbone HSQC peaks originating from Fc (16) and sFcγRIIIb (15), enabling us to map the spectral perturbation on their crystal structures (27, 28). The results showed that the interactions with HSA and polyclonal IgG-Fab cover these molecular surfaces extensively (**Figure 5**). Thus, the NMR data provide valuable information regarding the interactions of rituximab with the serum proteins, although the method required much higher concentrations of ^15^N-labeled proteins as compared with the expected concentration in the blood for rituximab treatment (>10 μg/mL), due to the sensitivity limitations of the NMR method (30). Human serum itself and the full-length form of serum polyclonal IgG caused more enhanced spectral changes of rituximab-Fc than the polyclonal IgG-Fab and HSA (**Figure 6**).

## 4 Discussion

This study demonstrated that HSA, the most abundant serum protein, negatively affects the interaction between rituximab and FcγRIIIa and compromises ADCC. The NMR data provide direct evidence for the interaction of HSA with rituximab, and to a lesser extent, with the extracellular region of FcγRIIIb. The extracellular region of FcγRIIIa shares 96% amino acid identity with that of FcγRIIIb and therefore possibly interacts with HSA to the same extent as FcγRIIIb in this condition, although their N-glycans may affect the interaction. HSA interacted with both the Fab and Fc regions of rituximab, but to a greater extent with Fab, which is not only responsible for antigen binding but also directly involved in the interaction with FcγRIIIa (13, 19, 31). Hence, HSA may interfere at various points in ADCC, at least for interaction with FcγRIIIa mediated by both Fab and Fc, and possibly for antigen recognition.

Residues perturbed by HSA were widely distributed in the Fc and sFcγRIIIb molecules, including their binding sites, suggesting that the interaction is not mediated by specific sites (**Figure 5**). Our previous NMR study demonstrated that mouse IgG2b-Fc interacted with polyclonal IgG-Fab from pooled human serum but to a considerably weaker extent with HSA (9). In contrast, analytical centrifugation detected weak reversible interactions between HSA and adalimumab, a human IgG1 monoclonal antibody antagonizing tumor necrosis factor (7). Thus, the interaction with HSA exhibits specificity for human IgG1 to a certain extent.

Although HSA harbors versatile binding pockets that accommodate basic, acidic, or neutral small molecules (32), the overall physical properties, such as net charges, rather than local structural features, may govern its interactions with macromolecules. Indeed, charge variation in IgG affects the binding to FcγRIIIa (33). In a neutral solution, HSA (pI = 5.7) is net negatively charged and therefore interacts preferably with positively charged proteins. This consistently explains the NMR observations, i.e., extensive, moderate, and undetectable interactions with rituximab-Fab (pI = 8.8), rituximab-Fc (pI = 7.1), and sFcγRIIIb (pI = 6.2), respectively. This may also explain why mouse IgG2b-Fc (pI = 6.3) shows little binding to HSA. This means that charge variations of IgG1, such as deamination, affect its interaction with HSA. According to this theory, an Fab with a λ chain would be more intensively interact with HSA than rituximab-Fab. This is because the Cλ domains have a higher isoelectric point (pI = 6.9 – 9.1) than that of the Cκ domain (pI = 5.6), which is harbored in rituximab-Fab.

Although much less pronounced than the effect of HSA, the Fab fragment from the pooled serum polyclonal IgG specifically interacted with the Fc region of rituximab and significantly affected ADCC. It was unexpected that polyclonal IgG-Fab interacted with the Fc region of rituximab, i.e. human IgG1-Fc, rather than the more heterologous rituximab-Fab harboring mouse-derived variable region. As in the HSA interaction, this significant preference may be attributed to the difference in net charge between Fab and Fc regions in rituximab because polyclonal IgG-Fab isolated from human serum generally has a pI of >7 (34). However, polyclonal IgG-Fab barely interacts with sFcγRIIIb, which has the lower pI value than rituximab-Fc, suggesting that some unknown factors determine the reactivity of polyclonal IgG-Fab. Anyway, the negative effect on ADCC is plausibly enhanced in the full-length form of serum polyclonal IgG with bivalency and bulkiness in addition to the Fc-mediated competition for FcγRIIIa (5). Indeed, the intact form of polyclonal IgG and human serum containing it had greater impacts on rituximab-Fc than the Fab fragment derived it (**Figure 6**).

Because many therapeutic antibodies and Fc-fusion therapeutics share the common Fc region derived from human IgG1 (35), serum polyclonal IgG as well as HSA are likely to interact with these protein drugs affecting their interactions with FcγRIIIa and functional efficacy. The interactions with the serum proteins cover the Fc surface so extensively that they can act pan-inhibitory on various Fc receptor-mediated functions and pharmacokinetics. Furthermore, the non-competitive inhibition of ADCC by HSA and polyclonal IgG-Fab suggests that they can be allosteric inhibitors of Fc affecting its dynamic structure (36). Therefore, controlling the interactions with the serum proteins is an essential factor to consider in the development of therapeutic antibodies and Fc-fusion therapeutics. Their interactions are likely to depend on artificial and naturally occurring modifications, such as drug conjugation and glycosylation, and are presumably controllable through mutational charge modifications. Serum components, including HSA and polyclonal antibodies, can differ qualitatively and quantitatively depending on the disease state and drug administration history (37). In this regard, methods to assess interactions with serum components are valuable for the development and application of antibody-based drugs with increased efficacy and safety.

## Data availability statement

The original contributions presented in the study are included in the article/supplementary material. Further inquiries can be directed to the corresponding author.

## Author contributions

SY: Conceptualization; data curation; funding acquisition; formal analysis; investigation; writing – original draft preparation; writing – review and editing. RY: Conceptualization; data curation; writing – review and editing. HY: data curation; writing – review and editing. MO: data curation; writing – review and editing. NW: data curation; writing – review and editing. YY: data curation; writing – review and editing. SU: data curation; investigation; writing – review and editing. KK: Conceptualization; funding acquisition; investigation; project administration; supervision; writing – original draft preparation; writing – review and editing. All authors contributed to the article and approved the submitted version.
